# IDENTIFYING UNMET NEEDS: THE RELATIONSHIP BETWEEN FRAILTY MEASURES AND PATIENT PRIORITIES CARE

**DOI:** 10.1093/geroni/igae098.2610

**Published:** 2024-12-31

**Authors:** Amy Thomas, Gwen Bernacki, Karl Brown, Terri May, Kirk Williams, Erica Martinez, Katherine Ritchey

**Affiliations:** VA Puget Sound Healthcare System, Seattle, Washington, United States; VA Puget Sound Healthcare System, Seattle, Washington, United States; VA Puget Sound Healthcare System, Seattle, Washington, United States; VA Puget Sound Healthcare System, Seattle, Washington, United States; VA Puget Sound Healthcare System, Seattle, Washington, United States; VA Puget Sound Healthcare System, Seattle, Washington, United States; VA Puget Sound Healthcare System, Seattle, Washington, United States

## Abstract

Patient Priorities Care (PPC) is an effective approach to identifying and aligning ‘what matters’ (i.e., values) with healthcare for patients with multimorbidity and frailty. VA metrics [i.e., Jen Frailty Index (JFI); Care Assessment Need (CAN)] predict mortality and institutionalization but may not correlate with the number and complexity of healthcare needs of veterans with multimorbidity. For patients referred to a VA Geriatric PPC Consultation clinic, recommendations and alignment of care were guided by a veteran-derived health outcome goal. Recommended changes to healthcare plans including rehabilitative or social service referrals were identified from electronic health records. Multiple linear regression was used to correlate total number of recommendations (outcome) with frailty (JFI) and multimorbidity (CAN) scores; interaction between frailty/multimorbidity scores with rurality was also explored. Rural/non-rural groups were demographically similar and had four recommendation subtypes per patient (mean). Median CAN was 85 (out of 99) and JFI 6 (out of 13). CAN or JFI scores had no effect on number of recommendations for both rural (p = 0.32 for CAN, p = 0.85, respectively) and non-rural (p = 0.09, p = 0.35, respectively) groups. There was no difference in the effect of CAN or JFI on the recommendations between rural vs. non-rural groups (p = 0.45, p = 0.43). JFI and CAN scores may not identify which older veterans have rehabilitative or social needs. Future work is needed to clarify how frailty and multimorbidity measures can guide referral to geriatric services and/or resource allocation to prevent disability.

